# Advances in Cardiovascular Biomarker Discovery

**DOI:** 10.3390/biomedicines8120552

**Published:** 2020-11-30

**Authors:** Crystal M. Ghantous, Layla Kamareddine, Rima Farhat, Fouad A. Zouein, Stefania Mondello, Firas Kobeissy, Asad Zeidan

**Affiliations:** 1 Department of Nursing and Health Sciences, Faculty of Nursing and Health Sciences, Notre Dame University-Louaize, Keserwan 72, Lebanon; cghantous@ndu.edu.lb; 2 Biomedical Sciences Department, College of Health Sciences, QU Health, Qatar University, Doha 2713, Qatar; lkamareddine@qu.edu.qa; 3 Biomedical and Pharmaceutical Research Unit, QU Health, Qatar University, Doha 2713, Qatar; 4 Department of Anatomy, Cell Biology and Physiology, Faculty of Medicine, American University of Beirut, Beirut 1107, Lebanon; rf57@aub.edu.lb; 5 Department of Pharmacology and Toxicology, Faculty of Medicine, American University of Beirut, Beirut 1107, Lebanon; fz15@aub.edu.lb; 6 Oasi Research Institute-IRCCS, 94018 Troina, Italy; stm_mondello@hotmail.com; 7 Department of Biomedical and Dental Sciences and Morpho-functional Imaging, University of Messina, 98125 Messina, Italy; 8 Department of Biochemistry and Molecular Genetics, Faculty of Medicine, American University of Beirut, Beirut 1107, Lebanon; firasko@gmail.com; 9 Department of Basic Medical Science, Faculty of Medicine, QU Health, Qatar University, Doha 2713, Qatar

**Keywords:** biomarkers, cardiovascular diseases, hypertension, proteomics, miRNA

## Abstract

Cardiovascular diseases are the leading causes of mortality worldwide. Among them, hypertension and its pathological complications pose a major risk for the development of other cardiovascular diseases, including heart failure and stroke. Identifying novel and early stage biomarkers of hypertension and other cardiovascular diseases is of paramount importance in predicting and preventing the major morbidity and mortality associated with these diseases. Biomarkers of such diseases or predisposition to their development are identified by changes in a specific indicator’s expression between healthy individuals and patients. These include changes in protein and microRNA (miRNA) levels. Protein profiling using mass spectrometry and miRNA screening utilizing microarray and sequencing have facilitated the discovery of proteins and miRNA as biomarker candidates. In this review, we summarized some of the different, promising early stage protein and miRNA biomarker candidates as well as the currently used biomarkers for hypertension and other cardiovascular diseases. Although a number of promising markers have been identified, it is unlikely that a single biomarker will unambiguously aid in the classification of these diseases. A multi-marker panel-strategy appears useful and promising for classifying and refining risk stratification among patients with cardiovascular disease.

## 1. Hypertension

Cardiovascular disease (CVD) refers to a group of disorders that includes hypertension, coronary artery disease, peripheral artery disease, stroke, congenital heart disease, and heart failure [1]. It is the leading cause of death worldwide, accounting for approximately 17.9 million deaths in 2016 alone [2]. The annual cost for the management of CVDs in the US in 2015 was an estimated $351.3 billion, accounting for the highest costing group in all diagnostic groups, and hypertension and its associated complications were responsible for more than 50% of deaths caused by CVDs [3]. 

Medications and health care services for hypertension cost the US approximately $51.2 billion in 2014 [4]. Being a CVD itself, hypertension is also a major risk factor for the development of other cardiovascular diseases, such as stroke, renal disease, and heart failure [5,6]. Clinically, hypertensive patients are divided into two groups: (1) stage 1 hypertension, where systolic/diastolic blood pressure consistently ranges between 140/90 and 159/99 mmHg [7], and (2) stage 2 hypertension, where systolic/diastolic blood pressure consistently exceeds 160/100 mmHg [8]. Based on its etiology, hypertension can be classified as either essential (primary) or secondary. Essential hypertension is the most common form of hypertension. Its occurrence is generally idiopathic with undefined mechanisms [6], but highly correlates with family history, sedentary lifestyle, salt intake, obesity, age, smoking, and stress [6,9]. Secondary hypertension, on the other hand, is directly linked to pre-existing pathophysiological disorders, such as renal disease, endocrine disorders, neurological diseases, and pregnancy [6,10].

Accurately diagnosing hypertension is not as simple as other diseases. For instance, diseases like cancer are assertively identified by tools like magnetic resonance imaging (MRI), histological-pathological examination, and molecular workup. However, blood pressure measurements may not be stable and elevated at all times. It is crucial to discover and use early stage biomarkers that indicate the early development of hypertension as well as other cardiovascular diseases before these diseases and their associated complications have already occurred. 

With chronic hypertension, the arteries undergo vascular remodeling. Their walls become stiffer and less elastic, thereby increasing the risk of vascular occlusion and rupture, and subsequently leading to organ damage or failure [11,12]. Among its manifestations, hypertension promotes vascular smooth muscle cell (VSMC) remodeling [12], endothelial cell dysfunction, and atherosclerosis [13].

### 1.1. Hypertension and Vascular Smooth Muscle Cell Remodeling 

VSMCs reside in the tunica media, the middle layer of blood vessels and the thickest layer in arteries. They contract and relax in response to different stimuli in order to regulate blood flow to the tissues that the vessels irrigate. In essential hypertension, small resistance arteries undergo vascular remodeling and become characterized by an increased wall thickness to lumen ratio and a narrower lumen [14,15]. 

**Figure 1:**
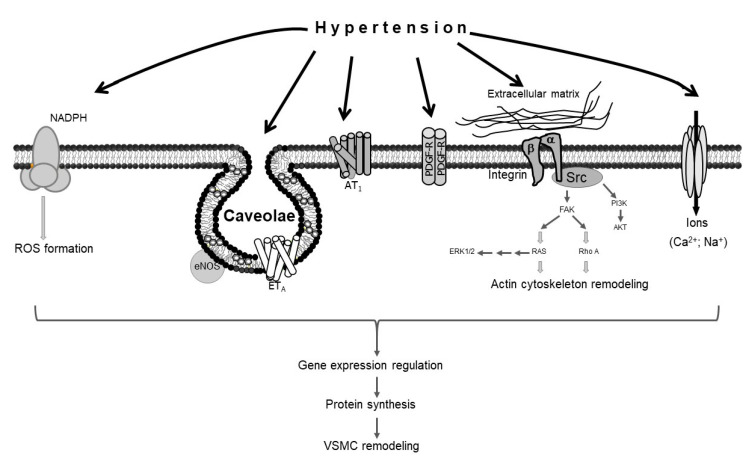
Schematic representation of vascular smooth muscle cell (VSMC) remodeling in response to hypertension. Hypertension stimulates different sensors in the plasma membrane of VSMCs, activating several signaling pathways that lead to VSMC remodeling.

Several molecular mechanisms mediate hypertension-induced vascular remodeling. The force of mechanical stretch exerted by hypertension on the vascular wall promotes the production of reactive oxygen species (ROS) [16], which in turn induce VSMC remodeling [17,18]. The excessive force of stretch mediated by hypertension also causes alterations in the extracellular matrix, activating the RhoA pathway, which in turn promotes actin cytoskeleton remodeling in VSMCs [16]; the hypertension-induced activation of extracellular signal-regulated kinases 1 and 2 (ERK1/2) and protein kinase B (AKT) also results in vascular remodeling [19,20]. Moreover, caveolae, which are lipid raft invaginations in the plasma membrane, mediate hypertension-induced VSMC modeling via endothelial nitric oxide synthase (eNOS) and endothelin receptor type A (ETA) [21,22,23]. Studies have also shown that angiotensin II type 1 receptor (AT_1_), platelet-derived growth factor receptor (PDGF-R), and specific ion channels, like voltage-gated calcium channels, are implicated in hypertension-induced VSMC remodeling [19,24,25,26,27] (Figure 1).

### 1.2. Hypertension and Endothelial Dysfunction

Endothelial cells are located in the tunica intima layer of blood vessels and form the luminal surface. Blood pressure exerts two types of forces on the endothelial cells: outward mechanical stretch and shear stress. When blood pressure is low, endothelial cells secrete a number of vasoactive molecules, like angiotensin II, endothelin-1, ROS, and prostanoids, which act on VSMCs to promote VSMC contraction and subsequent vasoconstriction [28,29]. In contrast, when blood pressure rises, vasodilator substances like nitric oxide (NO), prostacyclin, and endothelium-derived hyperpolarizing factor are produced by endothelial cells [30,31]. 

The forces exerted by hypertension cause endothelial damage and dysfunction, resulting in reduced production of NO [32,33]. Consequently, blood pressure-induced vasodilation is compromised. Moreover, hypertension-mediated endothelial dysfunction promotes the development of atherosclerosis.

Atherosclerosis is associated with the build-up of an atheromatous plaque, which is mainly composed of oxidized low-density lipoprotein (LDL) and macrophages inside the walls of arteries. It is a risk factor for coronary artery disease, myocardial infarction (MI), hypertension, stroke, and peripheral artery disease [34,35,36,37]. Arterial calcification is associated with atheroma progression and alters the mechanical properties of the vascular wall, thereby increasing the risk of rupture of the atherosclerotic plaque [38]. Discovering distinctive biomarkers that indicate early atherosclerosis development may allow the early detection of atherosclerosis, which in turn would encourage the patient to make healthy lifestyle changes or begin treatment in order to prevent its progression.

## 2. Biomarkers 

The discovery of biomarkers has become an essential and vibrant field in biomedical and clinical research. Biomarkers are objectively measured and used to indicate a certain biological state, whether physiological, pathological, or pharmacological [39]. Moreover, they can provide information about normal molecular physiology as well as disease activity and progression. They are also used by pharmacologists to gain insight into the mechanistic action of drugs and their efficacy, safety, and off-target actions [40]. Some biomarkers could be risk factors themselves and therefore potential targets of therapy [41,42]. Biomarkers may be found in biofluids, such as the blood and urine, and tissues (biosample), as well as recorded using tests like the electrocardiogram [43]. In the following sections, we outline the roles of validated blood-based markers for CVDs and discuss and provide perspectives on the emerging candidates, which include proteins and microRNAs (miRNAs).

### 2.1. Classical Biomarkers of Cardiovascular Disease

Obesity, smoking, hypertension, gender, age, LDL cholesterol, diabetes, and sedentary lifestyle are well-known risk factors for CVD development. However, these factors can only be used to pinpoint patients at high risk but never prevent or predict an acute or fatal attack, such as MI. Some of these factors have been used in the Framingham Risk Score [44], an algorithm that calculates the 10-year risk of developing cardiovascular adverse events. Low risk individuals have a score of less than 10%, while intermediate risk is shown at 10-20%, and high risk is seen when the score is over 20% [44].

There are currently several clinical biomarkers that are associated with cardiovascular events. These biomarkers include: C-reactive protein (CRP), cardiac troponins I and T (cTnI and cTnT), B-type natriuretic peptides (BNP and NT-proBNP), and D-dimer [45,46,47] (Summarized in Table 1). CRP is a pattern recognition molecule that is elevated in inflammatory conditions, such as atherosclerosis. CRP levels predict cardiovascular morbidity [48], and elevated CRP levels are directly correlated with future cardiovascular risks [49]. The cardiac troponins cTnI and cTnT are particularly significant biomarkers in diagnosing acute MI and in stratifying risks in acute coronary syndrome [50,51]. The B-type natriuretic peptides (BNP and NT-proBNP) are used as biomarkers to diagnose heart failure in both acute and chronic states [52]. D-dimer is a biomarker of thrombosis, cardiovascular mortality, acute aortic dissection, and ischemic heart disease [45,53,54]. Although these biomarkers are routinely used in clinical practice and have helped doctors save lives, they detect cardiovascular events after an attack has already occurred (late stage biomarkers). The challenge is to find biomarkers that detect early stage CVD in order to significantly reduce morbidity and mortality associated with cardiovascular events and improve prognosis.

### 2.2. Early Stage Biomarkers of Cardiovascular Disease

Discovering early stage biomarkers of CVD is crucial in predicting future cardiovascular events in both healthy and unhealthy individuals. Protein profiling using proteomic techniques and mRNA screening utilizing microarray platform and RNA sequencing allow the identification of dysregulated proteins and differentially expressed genes in early stages of disease. Table 2 lists some important early stage biomarkers of CVDs. With these biomarkers, biological mechanisms of CVDs can be better understood, and the prognoses of CVDs can be improved. For instance, Ceholski et al. have reported that lethal dilated cardiomyopathy and heart failure can be detected by a dominant Arg->Cys mutation at residue 9 in the phospholamban gene (PLN-R9C) [61]. Thus, PLN-R9C has the potential of serving as an early stage biomarker for cardiomyopathy and subsequent heart failure.

Another early detection biomarker is myeloperoxidase (MPO), an enzyme that catalyzes the formation of hyperchlorite from chloride and hydrogen peroxide (Table 2). It is secreted by active macrophages and neutrophils during an inflammatory process [62,63]. MPO and metalloproteases break down the collagen layer in an atherosclerotic plaque, thus leading to its erosion and rupture. High MPO levels are considered an early detection biomarker of CVD due to their correlation with atheroma instability. Clinical trials have found that elevated MPO levels are early indicators of coronary artery disease [64] even before detection by angiography or cardiac troponin levels [62]. However, MPO is not necessarily specific to CVD because macrophage and neutrophil activation can also occur in response to infections and inflammatory responses unrelated to the cardiovascular system [62].

Secreted frizzled related proteins (sFRPs) are secreted at the early stages of MI and function as *Wnt* antagonists [65] (Table 2). The *Wnt* pathway physiologically plays a role in cytoskeleton regulation and β-catenin stabilization, which in turn translocates to the nucleus to activate the gene expression that imposes an anti-apoptotic phenotype. When the *Wnt* pathway is antagonized by sFRP3, a pro-apoptotic pathway typical of MI and heart failure is activated [65]. Thus, MI, heart failure, and its adverse outcomes are associated with high circulating levels of sFRP3 [66], suggesting a potential role for sFRP3 as an early stage diagnostic biomarker of heart failure. Although there are more biomarkers that have been reported to be associated with CVD development (Table 2), the discovery of novel gene/protein biomarkers is still necessary to improve the prognostic accuracy of CVDs.

### 2.3. Second-Generation Biomarkers of Cardiovascular Disease

New research is directed at discovering second-generation biomarkers for CVD. Among them, miRNAs have been examined as potential biomarkers for several diseases, including cancer and neurodegenerative diseases [78,79]. However, their use in the cardiovascular field is relatively recent. A recent PubMed search for “miRNAs and human cardiovascular disease” resulted in 6780 hits versus “protein biomarkers and human cardiovascular disease”, which resulted in 64,902 hits (PubMed search Keywords: biomarkers, cardiovascular disease, human, miRNAs (or microRNAs), protein; November 2020). Thus, miRNAs are emerging as biomarkers in the areas of CVD with promising potential [80,81].

MiRNAs are short, non-coding oligonucleotides (20–26 nucleotides) that function to silence mRNAs and thus inhibit the translation of mRNAs to proteins [82,83]. They move from one cell to another in a process of intercellular communication to silence specific mRNAs in the target cell. MiRNAs circulate in the body in membrane-derived vesicles, such as microvesicles, exosomes, and apoptotic bodies, as well as bound to RNA-binding proteins, like Argonaute 2 protein (AGO2), or by high-density lipoprotein (HDL) [82,84,85,86,87]. The role of miRNAs has been recently proposed as next-generation biomarkers due to their integral role in mediating cellular and molecular functions.

Circulating miRNAs have emerged as biomarkers for several reasons. First, they are stable and resistant to changes in pH, temperature, freeze-thaw cycles, and long-term storage. Second, their sequences are generally conserved in different species. Third, several methods can be used to measure their levels, which have become correlated with different states of normal biological function as well as disease [88,89,90].

MiRNAs can be considered as reliable biomarkers for CVD. Table 3 summarizes some of the studied circulating miRNA biomarkers associated with CVD. For instance, miR-208 has been examined in the case of myocardial injury [91,92]. MiRNA array revealed that miR-208 is produced exclusively by the myocardium, and studies using real-time PCR confirmed that miR-208 levels in the plasma were significantly associated with myocardial injury similarly to cTnI [91], an already established biomarker of myocardial injury. Moreover, in patients with MI, the plasma levels of miR-208b and miR-499 were significantly elevated compared to the control and healthy individuals, and both were directly associated with cTnT and creatine phosphokinase [88]. Thus, miR-208b and miR-499 are potential candidate biomarkers for acute MI (Table 3). Interestingly, studies have shown that potential clinical confounders such as age, gender, renal function, and body mass index appear to not affect circulating miRNA levels [88], another aspect that makes miRNAs very appealing markers.

In addition, dysregulation of miR-21, let-7, miR-221, miR-27b, miR-222, miR-126, and miR-130a has been implicated in atherosclerosis, angiogenesis, and coronary artery disease [93,94,95,96] (Table 3). MiRNAs are also being studied in children to detect potential future events of CVD. Dysregulation of miRNA expression has been reported in congenital heart defects, coronary artery disease, and cardiometabolic disorders [97,98,99,100,101] (Table 3).

## 3. “Omics” and Systems Biology

Current research is directed at discovering new ideal biomarkers for CVDs. One of the fastest and most efficient approaches employs the recently thriving “omics” techniques. The “omics” universe uses novel technologies to make measurements in the fields of genomics, transcriptomics, proteomics, and metabolomics. They detect DNA, RNA, proteins, lipids, and metabolites that are expressed in different organ systems, such as the cardiovascular system, by using plasma, urine, whole blood, and tissues. The omics approach provides extensive amounts of data at all levels of biological function, from the sequence and expression of genes to the expression patterns of proteins and metabolites [109]. These technologies allow the identification of various molecules that are involved in normal physiological function as well as pathological events and their use as biomarkers [110].

### 3.1. Omics

Omics studies allow samples to be analyzed as a global set of macromolecules. The acquired data do not focus on one specific target, but rather on a whole set of molecular species. This is the main aspect of systems biology, which connects molecules and their interactions to the function as a whole in the living system [111]. When the interconnections between pathways are deciphered, a universal physiological system can be deduced. As a result, predictions can be made about biological responses to certain abnormalities, such as environmental interventions and disease, thus providing information on the development, prevention, prediction, and treatment.

Omics techniques can generate up to thousands of results at once. This remarkable feature is attributed to their ability to detect even thousands of molecules and expression patterns in every experimental run and in a short time frame. As such, potential biomarkers of CVD can be easily identified. However, validation of these biomarkers using different methods is essential and necessary before translating these biomarkers from the lab into the clinic.

Proteomics profiles the expression and function of the protein complement on a global scale [112], providing a rapid and precise way to discover and identify proteins that are differentially expressed and to characterize certain disease states. Studying the proteome of injured, abnormal cardiovascular tissue allows researchers to identify biomarkers of disease development, progression, and treatment. Cardiomyopathy [113], myocardial ischemia [114], cardiac hypertrophy [115], and heart failure [116] have been analyzed using proteomic-based studies. However, the use of proteomics to discover biomarkers in CVD is still developing, but promising to be a very useful tool.

### 3.2. Proteomic Advances in Detecting Vascular Diseases

Atherosclerosis is usually silent until it evolves into a detrimental stage leading to stroke, MI, or peripheral artery disease, thus necessitating an early predictor marker that can detect these indications prior to being fully apparent. Previously, the standard method of studying atherosclerosis was to focus on a certain protein believed to play a role in the development or progression of atheromas. This approach, although targeted, is time-consuming and only focuses on one protein at a time. On the other hand, proteomic platforms allow a multitude of proteins potentially involved in atherosclerosis development to be identified and analyzed at once.

Comparing the differential expression of proteins in atherosclerotic plaques and affected arteries with non-affected arteries allows the detection of biomarkers involved in atherosclerosis, and as such are analyzed in biological fluids like plasma or urine. For example, haptoglobin and serum amyloid-A overexpression have been associated with atherothrombotic ischemic stroke, as studied by matrix-assisted laser desorption/ionization-time-of-flight (MALDI-TOF) mass spectrometry (MS) [60]. These findings were validated using enzyme-linked immunosorbent assay (ELISA) techniques [60]. Thus, levels of haptoglobin and serum amyloid A can be considered as biomarkers to predict atherothrombotic ischemic stroke as opposed to cardioembolic stroke (Table 1).

Another protein identified by proteomic studies is β 2-microglobulin (Table 2). Surface-enhanced laser desorption/ionization-TOF (SELDI-TOF)-MS revealed that β 2-microglobulin protein was significantly higher in the plasma of patients suffering from peripheral arterial disease with high prognostic values [74], as validated and confirmed by ELISA [74].

Several other proteins associated with unstable human carotid plaques have also been detected and classified as potential biomarkers. Topoisomerase-II-α, caspase-9, junctional adhesion molecule-1 (JAM-1), Grb2-like adaptor protein (GADS), and TNF receptor-associated factor 4 (TRAF4) were found to be over-expressed in VSMCs, endothelial cells, and infiltrated macrophages [117]. G-protein-coupled receptor kinase-interacting protein (GIT1), c-src, and c-jun N-terminal kinase (JNK) were also upregulated in atherosclerotic plaques [117]. The discovery of early stage biomarkers in detecting and profiling CVD could be used as a prophylactic measure to prevent and reverse disease progression.

## 4. Biomarkers Reflecting Hypertension Pathogenesis

Since hypertension promotes the development of other CVDs, identifying antecedent, screening, and early stage diagnostic biomarkers is crucial in preventing hypertension-associated CVDs. Biomarkers of hypertension include those that indicate oxidative stress and inflammation since hypertension is associated with these states. Interestingly, adipokines have also emerged as potential biomarkers of hypertension. The following section describes these biomarkers in detail.

### 4.1. Biomarkers Reflecting Oxidative Stress

Hypertension is highly associated with oxidative stress, which in turn mediates hypertension-induced cardiovascular complications [16,118]. Several molecules have been shown to reflect the oxidative state. For instance, measurements of nitrite (NO_2_^-^) and nitrate (NO_3_^-^) can be used because they are markers of NO bioavailability. NO_2_^-^ and NO_3_^-^ are products of oxidative degradation of NO, which physiologically causes vasodilation and prevents hypertension [119,120] (Table 4). Their increased levels indicate a reduction in NO bioavailability and are thus associated with hypertension.

Asymmetric dimethylarginine (ADMA) and uric acid are other biomarkers for hypertension (Table 4). They inhibit the production of NO [121,122], so their increased levels are associated with reduced NO bioavailability and impaired vasodilation [123,124,125]. Moreover, ADMA levels are correlated with acute coronary events and can also be used as a biomarker for adverse cardiac outcomes [126]. ROS are another indicator of hypertension, since hypertension has been shown to directly increase ROS [16,118] (Summarized in Table 4). Although the aforementioned factors are widely used for the diagnosis of hypertension, there is an increasing demand to introduce more biological markers such as proteins and genes to improve the prediction of this condition. According to the Rat Genome Database, hundreds of genes are associated with hypertension (Rat: https://rgd.mcw.edu/rgdweb/elasticResults.html?term=hypertension&chr=ALL&start=&stop=&species=Rat&category=Gene&objectSearch=true; human: https://rgd.mcw.edu/rgdweb/elasticResults.html?term=hypertension&chr=ALL&start=&stop=&species=Human&category=Gene&objectSearch=true).

### 4.2. Protein Biomarkers Reflecting Inflammation

Since hypertension is associated with vascular inflammation [130], markers of inflammation can be used as biomarkers for hypertension. The cell adhesion molecules vascular cell adhesion molecule (VCAM), intercellular adhesion molecule (ICAM), and platelet endothelial cell adhesion molecule (PECAM) allow inflammatory cells to adhere to the vascular wall [131,132,133]. High plasma levels of these molecules have been shown to be associated with hypertension [132,133,134].

Hypertensive patients have higher circulating levels of the inflammatory cytokines IL-1β, IL-10, and tumor necrosis factor-alpha (TNF-α), indicating the potential use of these inflammatory biomarkers as markers for hypertension [135]. Elevated levels of IL-1β, IL-10, and TNF-α are also correlated with increased arterial stiffness associated with hypertension [135]. Moreover, IL-6 and TNF-α levels can be used as independent risk factors for hypertension in healthy individuals [136] (Summarized in Table 5).

### 4.3. Adipokines as Biomarkers of Hypertension

We and others have shown the significant association between the hormone leptin and hypertension [16,144,145,147,148,149]. Leptin is an obesity-associated adipokine that physiologically reduces appetite and increases energy expenditure. Research has shown that hypertensive patients have higher circulating leptin levels [150] and that leptin can be used as a predictor of new-onset hypertension [144]. Moreover, a recent biomedical and proteomics study conducted in our lab has shown that leptin is produced by VSMCs and that its synthesis is upregulated by hypertension (unpublished data and [16]). In turn, leptin contributes to VSMC hypertrophy and promotes atherosclerosis [16,151] (Summarized in Table 5).

Adiponectin is another adipokine that is emerging as a biomarker for hypertension [152] (Table 5). This anti-inflammatory protein has been shown to exert cardioprotective effects on the heart by inhibiting pressure overload-induced cardiac hypertrophy and protecting against myocardial injury after ischemia-reperfusion [153,154,155]. Studies have shown that circulating adiponectin levels are reduced in hypertensive patients [146], which may explain the detrimental effects of hypertension on the cardiovascular system. In addition, we have recently shown that adiponectin is not only expressed by adipocytes, but also VSMCs, and that adiponectin supplementation reduces hypertension-induced VSMC hypertrophy [17,156].

## 5. Conclusions

CVDs pose a huge global health and economic burden. They are the leading cause of death worldwide, and hypertension and its complications are responsible for an extremely high mortality rate associated with CVDs. Using biomarkers, namely early stage biomarkers of CVD, could potentially save many lives and help us win the fight against CVDs, all in the field of preventative medicine. The ideal biomarkers of CVD and hypertension or susceptibility to their development can be defined by alterations in a specific indicator’s level/concentration between healthy individuals and patients. These include changes at the level of proteins, genes, and miRNAs. The identification of these molecules is facilitated with the use of proteomic approaches, such as MALDI-TOF and SELDI-TOF, allowing global profiling of the protein complement. The use of microarrays and miRNA sequencing for miRNA expression profiling have also given rise to the discovery of many miRNAs that could be used as biomarkers for CVD. In this review, we summarized different protein and miRNA biomarker candidates for hypertension as well as other CVDs.

It is very important that a biomarker be specific and sensitive to a certain disease. In the case of CVDs, many of the biomarkers used clinically are late stage biomarkers, indicating the presence of a disease that has already developed. Identifying early stage biomarkers of CVDs is of utmost importance in preventing these diseases from progressing and inducing their associated complications.

Proteomics is a promising tool for the discovery of new biomarkers. The use of new technology allows scientists to discover and validate new biomarkers related to the early progression of hypertension as well as other CVDs [110,115]. These new proteomic tools provide many advantages; first, they require a small volume of sample, and second, they can detect the effect of hypertension on several proteins simultaneously between samples. On the other hand, these methods require the presence of appropriate and specific antibodies for targeted biomarkers. MiRNAs have also emerged as potential diagnostic biomarkers for CVD. Unfortunately, measuring and comparing miRNA concentrations in body fluid samples is difficult due to the low levels of miRNAs. This will be a critical challenge in the near future.

Although a number of promising markers have been identified (Table 1, Table 2, Table 3, Table 4 and Table 5), it is unlikely that a single protein biomarker will unambiguously aid in the classification of normotensive and hypertensive patients as well as those suffering from other CVDs. A multi-marker panel-strategy appears as a useful and promising approach for classifying and refining risk stratification among patients with CVDs. Moreover, many biomarkers have already been identified and are medically used, but their use in the clinic has been limited to post-injury or post-attack. The challenge is not only limited to finding the best panel of biomarkers, but implementing their use in the clinic in a timely and cost-effective manner.

## Figures and Tables

**Table 1:** Late stage protein biomarkers of cardiovascular disease.

Proteins Associated with CVD	Function/Description	Type of CVD They Help Diagnose	Levels in CVD	Reference(s)
C-reactive protein (CRP)	Pattern recognition molecule that is increased in inflammation or tissue injury	Inflammatory conditions (atherosclerosis)	Elevated	[55,56]
Cardiac troponin I (cTnI)	The subunit of troponin that binds to actin and maintains the troponin-tropomyosin complex	Acute myocardial infarction and acute coronary syndrome	Elevated	[50]
Cardiac troponin T (cTnT)	The subunit of troponin that binds to tropomyosin to form a troponin-tropomyosin complex	Acute myocardial infarction and acute coronary syndrome	Elevated	[50]
B-type natriuretic peptides (BNP and NT-proBNP)	Reduces plasma volume and blood pressure	Ventricular hypertrophy and acute and chronic heart failure	Elevated	[52]
D-dimer	Fibrin degradation product from fibrinolysis of blood clots	Thrombosis, ischemic heart disease, acute aortic dissection, cardiovascular mortality	Elevated	[46,53]
Tetranectin	Binds to kringle 4 of circulating plasminogen, upregulating the activation of plasminogen into plasmin in fibrinolysis	Presence and severity of diseased coronary arteries	Elevated	[57]
Serum cyclin-dependent kinase 9	Regulation of cell cycle and activation of inflammatory response genes	Atherosclerotic inflammation	Elevated	[58]
Endogenous ouabain	Glycoside that inhibits the Na^+^/K^+^-ATPase	Heart Failure	Elevated	[59]
Haptoglobin	Acute phase protein that binds to hemoglobin and also has antioxidant activity	Atherothrombotic ischemic stroke	Elevated	[60]
Serum amyloid A	Acute phase protein that increases the expression of pro-thrombotic and pro-inflammatory molecules	Atherothrombotic ischemic stroke	Elevated	[60]

**Table 2:** Early stage protein biomarkers of cardiovascular disease.

Proteins Associated with CVD	Function/Description	Type of CVD They Help Diagnose	Changes/Levels in CVD	Reference(s)
Phospholamban	Regulates cardiac contractility by inhibiting sarco/endoplasmic reticulum calcium transport ATPase (SERCA) in its dephosphorylated form	Early onset of dilated cardiomyopathy and heart failure	Dominant Arg-> Cys mutation at residue 9 (loss-of-function mutation)	[61]
Myeloperoxidase (MPO)	- Catalyzes the formation of hyperchlorite from chloride and hydrogen peroxide - Bactericidal agent produced by monocytes and activated neutrophils - Promotes oxidation of LDL and oxidative modification of apolipoprotein A-I	Unstable atheroma, coronary artery disease, ischemic heart disease, stroke	Elevated	[67,68,69]
Secreted frizzled related proteins (sFRPs)	Modulate Wnt signaling	Myocardial infarction and heart failure	Elevated	[65,66]
Serum amyloid A	Acute phase protein that increases the expression of pro-thrombotic and pro-inflammatory molecules	Coronary artery disease, atherosclerotic plaque destabilization, acute aortic dissection	Elevated	[70,71,72,73]
β 2-microglobulin	Membrane protein that associates with heavy chains of class I major histocompatibility complex proteins	Peripheral arterial disease	Elevated	[74]
Junctional adhesion molecule A (JAM-A)	Regulates tight junction permeability and integrity of endothelial and epithelial cells	Acute endothelial activation and dysfunction	Elevated	[75]
Platelet/endothelial cell adhesion molecule-1 (PECAM-1)	Transduces mechanical signals in endothelial cells and regulates migration of leukocytes through the endothelium	Acute coronary syndromes	Elevated	[76]
Vitamin D-binding protein (VTDB)	Binds to vitamin D and its plasma metabolites and transports them to target tissues	Coronary artery stenosis	Reduced	[77]

**Table 3:** Non-protein biomarkers of cardiovascular disease.

Non-Proteins Associated with CVD	Type of CVD They Help Diagnose	Changes/Levels in CVD	Reference(s)
miR-208b and miR-499	Acute myocardial infarction	Elevated	[88,91]
miR-21, miR-130a, miR-27b, and miR-210	Atherosclerosis obliterans and peripheral arterial disease	Elevated	[102]
miR-221 and miR-222	Atherosclerosis obliterans and peripheral arterial disease	Decreased	[102]
miR-34a, miR-21 and miR-23a	Coronary artery disease	Elevated	[103]
miR-26a	Hypertension	Decreased	[104]
miR-29a	Obstructive cardiomyopathy	Elevated	[105]
miR-29c	Aortic stenosis	Elevated	[105]
miR-499 and miR-133a	Myocardial infarction	Elevated	[106]
miR-1, miR-208a, and miR-499	Myocardial ischemic reperfusion injury	Elevated	[107]
miR-223	Acute ischemic stroke	Elevated	[108]

**Table 4:** Molecules used as biomarkers of hypertension.

Molecule	Function/Description	Levels in Hypertension	Reference(s)
Nitrate and nitrite	Physiological reservoir of NO that can be reduced to NO to regulate signal transduction	Elevated	[120,127]
Asymmetric dimethylarginine (ADMA)	Inhibits nitric oxide synthase	Elevated	[123]
Reactive oxygen species (ROS)	Highly reactive signal transduction molecules that cause nucleic acid, lipid, and protein damage when present in high concentrations (oxidative stress)	Elevated	[118,128]
Uric acid	Final oxidation product of purine metabolism	Elevated	[125,129]

**Table 5:** Inflammatory biomarkers of hypertension.

Inflammatory Mediators	Function/Description	Levels in Hypertension	Reference(s)
Vascular cell adhesion molecule (VCAM)	Endothelial cell surface glycoprotein that allows endothelial cell-leukocyte adhesion in inflammation	Elevated	[137]
Intercellular adhesion molecule (ICAM)	Endothelial cell surface glycoprotein that aids in endothelial cell-leukocyte adhesion	Elevated	[138]
Platelet endothelial cell adhesion molecule (PECAM)	Cell surface protein of platelets, monocytes, neutrophils, subsets of T cells that aids in leukocyte transendothelial migration, and a constituent of the endothelial intercellular junctions	Elevated	[132]
6-keto-prostaglandin F1a	Stable and active metabolite of prostacyclin that promotes vasodilation and inhibits platelet aggregation	Reduced	[139]
C-reactive protein (CRP)	Activates complement and binds to foreign and damaged cells and tissue	Elevated	[140]
Tumor necrosis factor (TNF-α)	Pro-inflammatory cytokine involved in apoptosis, cell proliferation, differentiation, and platelet activation	Elevated	[136,141]
IL-10, IL-1β	IL-10: Cytokine involved in mediating the inflammatory response, B cell survival, proliferation and antibody production, and nuclear factor kappa-light-chain-enhancer of activated B cells (NF-κB) activity IL-1β: Cytokine involved in regulating the inflammatory response, cell proliferation, differentiation, apoptosis, and cyclooxygenase-2 induction	Elevated	[135]
IL-6	Immune response in inflammation	Elevated	[136]
P-selectin	Cell adhesion molecule of platelets and endothelial cells that works in the interaction of leukocytes with platelets or endothelial cells	Elevated	[142]
Oxidized-LDL	Taken up by macrophages to form foam cells, a key step in atherosclerosis development	Elevated	[139]
Renin and prorenin	Renin hydrolyzes angiotensinogen to angiotensin I, while prorenin is its inactive precursor	Elevated	[143]
Leptin	- Hormone mainly produced by adipocytes that acts as a satiety factor to increase energy expenditure by signaling at the hypothalamus - Promotes VSMC hypertrophy - Pro-inflammatory cytokine - Regulates puberty, menstrual cycles, and reproductive function	Elevated	[144,145]
Adiponectin	- Insulin-sensitization and fatty acid oxidation - Anti-inflammatory - Cardioprotective	Reduced	[146]
